# A Study of Eighteen Consecutive Cases of Whooping-Cough Treated by the Elastic Abdominal Belt

**Published:** 1905-03

**Authors:** 


### SELECTIONS AND ABSTRACTS.

---

A Study of Eighteen Consecutive Cases of Whooping-Cough Treated by the Elastic Abdominal Belt.

This method consists in the application of a slightly constricted belt to the abdomen of all children having pertussis. The method of applying the belt is as follows: A stockinette band is placed on a baby with whooping-cough in the same manner as is done by orthopedists before applying the plaster-of-paris jacket. This band extends from the axilla to the pubes, and fits the baby snugly. Two muslin shoulder-straps are used to prevent the band from slipping down. On this stockinette band a single width of silk elastic bandage is sewn, extending entirely around the body and covering the abdomen. This silk elastic bandage is of the same quality as that used for elastic stockings. If the child is under one year old, it will be found necessary to use but one width of this elastic bandage; in an older child two widths will often be found necessary to cover the entire abdomen. This silk elastic bandage is pinned in place when very slightly on the stretch. After it is pinned in place it should be sewn to the stockinette band underlying it all around its entire edge. This procedure keeps the silk elastic belt flat and prevents its rolling up or becoming creased. The lower projecting portion of the stockinette should be pinned down to the outside of the diaper or other clothing, thus keeping the elastic belt smooth over the abdomen.

The benefit of the belt is seen in cutting short of the vomiting spells. The child will cough, but he will not vomit. The final results are as follows:

1. Out of eighteen cases there have been but six failures to benefit cough.

2. Out of eighteen cases there has been but one failure to benefit vomiting. Positive good effects on coughing is seen in 66 per cent. of cases in series.—*T. W. Kilmer, Journal of the American Medical Association.*

